# Characterization of an Algorithm for Autonomous, Closed-Loop Neuromodulation During Motor Rehabilitation

**DOI:** 10.1177/15459683241252599

**Published:** 2024-05-07

**Authors:** Joseph D. Epperson, Eric C. Meyers, David T. Pruitt, Joel M. Wright, Rachael A. Hudson, Emmanuel A. Adehunoluwa, Y-Nhy Nguyen-Duong, Robert L. Rennaker, Seth A. Hays, Michael P. Kilgard

**Affiliations:** 1 Texas Biomedical Device Center, The University of Texas at Dallas, Richardson, TX, USA; 2 Department of Bioengineering, Erik Jonsson School of Engineering and Computer Science, The University of Texas at Dallas, Richardson, TX, USA; 3 Department of Neuroscience, School of Behavioral and Brain Sciences, The University of Texas at Dallas, Richardson, TX, USA

**Keywords:** closed loop neurostimulation, neuroplasticity, rehabilitation, stroke, spinal cord injury, telerehabilitation, vagal nerve stimulation, vagus nerve stimulation

## Abstract

**Background:**

Recent evidence demonstrates that manually triggered vagus nerve stimulation (VNS) combined with rehabilitation leads to increased recovery of upper limb motor function after stroke. This approach is premised on studies demonstrating that the timing of stimulation relative to movements is a key determinant in the effectiveness of this approach.

**Objective:**

The overall goal of the study was to identify an algorithm that could be used to automatically trigger VNS on the best movements during rehabilitative exercises while maintaining a desired interval between stimulations to reduce the burden of manual stimulation triggering.

**Methods:**

To develop the algorithm, we analyzed movement data collected from patients with a history of neurological injury. We applied 3 different algorithms to the signal, analyzed their triggering choices, and then validated the best algorithm by comparing triggering choices to those selected by a therapist delivering VNS therapy.

**Results:**

The dynamic algorithm triggered above the 95th percentile of maximum movement at a rate of 5.09 (interquartile range [IQR] = 0.74) triggers per minute. The periodic algorithm produces stimulation at set intervals but low movement selectivity (34.05%, IQR = 7.47), while the static threshold algorithm produces long interstimulus intervals (27.16 ± 2.01 seconds) with selectivity of 64.49% (IQR = 25.38). On average, the dynamic algorithm selects movements that are 54 ± 3% larger than therapist-selected movements.

**Conclusions:**

This study shows that a dynamic algorithm is an effective strategy to trigger VNS during the best movements at a reliable triggering rate.

## Introduction

Neurological injuries are a common cause of disability in the U.S. There are approximately 800 000 strokes each year and over 300 000 people live with the effects of spinal cord injury (SCI).^1,2^ Many survivors are left with long-term upper limb hemiparesis, which can lead to disability.^3^ There is a clear and present need to develop interventional strategies to reduce this disability.

Recently, a strategy based on delivering bursts of vagus nerve stimulation (VNS) concurrent with rehabilitation received Food and Drug Administration (FDA) approval for the treatment of upper extremity motor deficits associated with chronic ischemic stroke.^4-6^ VNS therapy is premised on the timing of VNS concurrent with upper limb movement during rehabilitative exercises.^7^ Limb movement is driven by engagement of motor networks in the central nervous system, and the concurrent VNS generates a rapid release of neuromodulators that facilitates synaptic plasticity in the active motor networks. Consequently, degradation of the timing between VNS and the occurrence of the target movement reduces the efficacy of this approach. Studies in animal models show that delaying VNS until after training results in significantly less recovery.^8^ Moreover, even VNS delivered during rehabilitative exercises fails to be effective if it is not delivered concurrent with the best movements.^9^ Finally, emerging clinical studies provide some preliminary evidence of the need for precise timing. Whereas VNS delivered by a therapist during movements produces robust enhancement of upper limb recovery, additional VNS delivered during unsupervised exercises where stimulation did not explicitly coincide with movement provides comparatively modest benefits.^5,6,10^

In addition to these lines of evidence, the excellent adherence to the use of VNS at home and compounding functional benefits raise the prospect that a strategy to allow precise timing of stimulation during movement holds promise to maximize the benefits of VNS.^10^ Telerehabilitation solutions promote therapy adherence after neurological injury and demonstrate equivalent or better outcomes when compared to conventional face-to-face therapy.^11^ Additionally, telerehabilitation increases engagement and permits longer courses of rehabilitation, which has been shown to produce additional recovery.^12,13^ Take-home systems, such as RePlay, have been developed to support high-repetition motor rehabilitation and could be readily combined with strategies to improve VNS stimulation timing. To this end, we sought to leverage advances in rehabilitative technology and use miniaturized sensors in conjunction with an algorithm to design a system that automatically triggers stimulation based on selected parameters of movement during rehabilitation. To develop the algorithm, we analyzed data previously collected from 14 stroke and 18 cervical SCI patients using motion controllers to perform rehabilitative exercises. We selected the relevant sensor dimension for each exercise to isolate a single signal, then allowed an algorithm to simulate when to deliver stimulation as the exercise progressed. We simulated the application of 3 different algorithms to the signal and analyzed their triggering choices. Based on this analysis, we determined that a dynamic algorithm reliably selected the best movements with desired timing intervals. The dynamic algorithm continually adapts over time to adjust for intermittent periods of rest and person-to-person variability and can flexibly be applied to signals from various controllers (handheld sensors and touchscreen) while maintaining similar triggering characteristics. In addition to the simulated triggering analyses, we compared the dynamic algorithm to actual manual VNS triggers selected by a therapist from the same dataset. Validation of this approach shows that the dynamic algorithm selects optimal movements comparable to or better than a trained human observer. These findings lay the groundwork for the implementation of this approach to supplement delivery of VNS in future studies.

## Methods

### Study Design and Testing Protocol

All procedures were approved by the Institutional Review Board at the University of Texas at Dallas.^14,15^ A total of 32 participants ages 23 to 77 years with a history of upper limb motor impairment due to stroke or SCI (mean time since neurological injury was 5.8 ± 1.4 years) were recruited for a VNS clinical study with RePlay, a tablet-based rehabilitation system,^16^ and ReStore, an implantable device for VNS.^17^ All participants had motor impairments in the upper limbs with some residual function. Participants with stroke mostly completed range of motion exercises and SCI participants primarily completed isometric strength exercises, according to their deficits. We pooled their data together to increase the number and type of unique exercises to analyze. Study participants used the FitMi handheld motion controller (Flint Rehab, California) and the ReCheck system to perform rehabilitative exercises while playing games on an Android tablet.^18^ The FitMi controller is a rubberized puck that contains several sensors including a 3-axis accelerometer/gyrometer, magnetometer, and a force sensor. The ReCheck system supports 4 isometric tasks and 3 range-of-motion tasks via interchangeable modules. In addition to the FitMi and ReCheck controllers, an additional game allowed use of the tablet’s touchscreen for gameplay. Physical therapists guided participants to perform exercises that challenged range of motion and isometric strength. Measurements from the sensors included rotation angle (°), pressing and pinching force (g), and movement distance. Measurements from the touch screen included speed of finger movements across the screen. The study contained both traditional repetition-based exercises and dynamic game-controlling movements, each measured by a sensor array housed in the selected handheld device. If patients were not able to grasp and hold the device, the device was affixed to a stabilizing base.

### Signal Processing

#### Movement Signal Capture and Preprocessing

**Figure 1.**
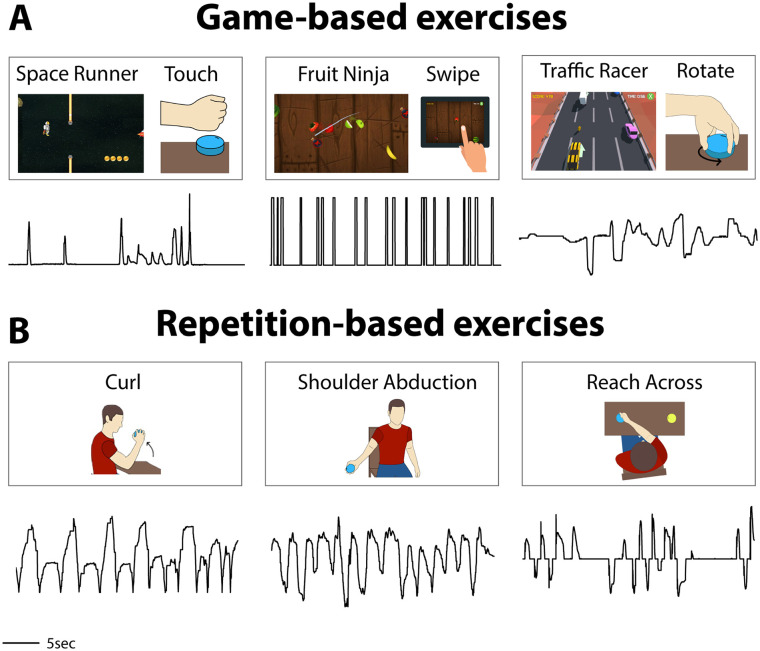
Examples of rehabilitative exercises and the corresponding movement signals collected from participants with stroke or SCI. Game titles and exercise types are listed above each representative signal. Participants controlled 9 sensing devices and 7 games during rehabilitative exercises to produce the movement signals in this study. Not all combinations are shown here. (A) Some exercises are conducted during gameplay. The Space Runner game responds to a force signal. Participants control the Fruit Ninja game with a touch screen, where they drag a finger across the touch screen to produce a signal that represents finger location over time. Here, the touch signal is shown as either swiping or not swiping. Participants control the Traffic Racer game by rotating a sensor puck that rests on a table. (B) Some exercises are performed without companion games to replicate traditional rehabilitation. A sensor puck can be used to detect movement for repetitive exercises such as curls, shoulder abduction, or reach across.

To capture movement data during rehabilitative exercises, the tablet application streamed and processed incoming 60 Hz data from the controllers and saved the data to local storage for offline analysis (Figure 1). Custom Python routines were developed for simulations and analysis.

Each algorithm accepted a preprocessed discrete signal of a single dimension. To select the relevant channel, RePlay required users to select specific exercises prior to movement initiation. This selection process guides the software to isolate the movement data to the specific sensor and dimension that matched the exercise. The software then processed the rotation or force signals to construct movement signals that describe rate of change over time. To begin, unprocessed movement signals were smoothed with a moving average filter (discrete, linear convolution, 300 ms of movement data in smoothing window, and kernel size varied with calculated sampling rate). About 300 ms of movement data was chosen for the smoothing window because large movements require approximately 300 ms to complete.^19^ At each point in time, the gradient of the most recent values of the smoothed movement signal was obtained to calculate the rate of change. The mean gradient value over the was calculated, resulting in a single value that represented the average rate of change over the 300 ms window. Each consecutive movement signal value was processed in the same manner. This signal processing resulted in a rate-of-change-based signal that could be used as input to an algorithm for stimulation (Figure 4). For force-based exercises, this signal indicates the rate of change of force, which may be positive (pressing or gripping) or negative (releasing). For rotation-based exercises, a positive signal indicates clockwise angular rate of change of the sensor puck, and a negative signal indicates a counterclockwise angular rate of change. For touchscreen data, this created signals that described the speed of touch over time.

We processed manual triggering data for comparison with the algorithms. A Google Pixel 2 smartphone triggered and recorded therapist-selected manual stimulations during the study, while a Samsung Galaxy Tab S4 tablet captured movement data during gaming and repetition-based exercises. To align clocks between devices, we calculated the average difference between session start time on the tablet and stimulation start time on the smart phone to realign the triggers with the movement data.

Movement minimums were selected for each exercise to separate movement from noise. The movement minimum for each exercise was determined by measuring the mean maximum value of the signal when a control subject held the controller with the arm at rest. Any activity that did not exceed the value of the movement minimum was excluded.

#### Dynamic Algorithm Process

As movement began and progressed, signal preprocessing was performed as described above, and each sample was passed to the algorithm. The algorithm placed each incoming sample into a buffer that held up to 3000 of the most recent samples. The buffer size was selected so that it was long enough to capture short game sessions (~1 minutes) but short enough to adjust to changes in movement amplitude that may happen due to fatigue or level changes in longer games (>4 minutes). Stimulation was prevented at the beginning of the game prior to movement initiation. The dynamic algorithm continuously analyzed the 3000-sample buffer and calculated a rate of change threshold value at the user-selected percentile and adjusted it for every new preprocessed sample. If the dynamic threshold was surpassed by an incoming value, the algorithm delivered a simulated VNS trigger. Optionally, users could set values for the minimum interstimulus interval and directionality.

#### Static Algorithm Process

As movement began and progressed, signal preprocessing was performed as described above, and each sample was passed to the algorithm. As each sample was analyzed, the static algorithm compared its value to a user-selected multiple of the movement minimum for that exercise. If the static threshold was surpassed by an incoming value, the algorithm delivered a simulated VNS trigger. Optionally, users could set values for the minimum interstimulus interval and directionality.

#### Periodic Algorithm Process

No signal preprocessing or continuous analysis were required for the periodic algorithm, as the nature of the algorithm is signal agnostic. Users were required to set a value for the interval between stimulations. The algorithm delivered a simulated VNS trigger periodically at the set interval.

### Statistics

Data are reported as mean ± standard error of the mean (SEM) or median with interquartile range (IQR). Where appropriate, standard parametric statistical tests (paired or unpaired *t*-tests) were used to make comparisons. Statistical tests for each comparison are noted in the text. Paired 2-tailed *t*-tests were used to determine differences in the triggering quality and rate of the algorithms and unpaired 2-tailed *t*-tests were used to determine differences in the movement signal pairings. The threshold for statistical significance was set at *P* < .05. Error bars in figures represent SEM. Whiskers in boxplots represent *Q*1 − (1.5 × IQR) or Q3 + (1.5 × IQR).

## Results

### Collection of Quantitative Upper Limb Movement Data From Stroke and SCI Patients

We sought to design a real-time algorithm that could be used to identify the best movements during a variety of different rehabilitative exercises and on average produce 5 stimulation pairings per minute, based on the most effective paradigms from preclinical studies. To develop this algorithm, we simulated various triggering algorithms on a previous set of rehabilitative movement data collected from 14 stroke and 18 SCI patients with impairments in upper limb motor function. The dataset includes captured movements from 9 sensing devices and 7 games. Data was collected from 1160 exercise sessions of 30 seconds or longer. Individual sessions had unique characteristics relative to each participant, game, exercise, and controller type (Figure 1). FitMi puck and ReCheck device movement was measured as rotation angle or force, and touch screen movement was measured as swipe speed.

**Figure 2.**
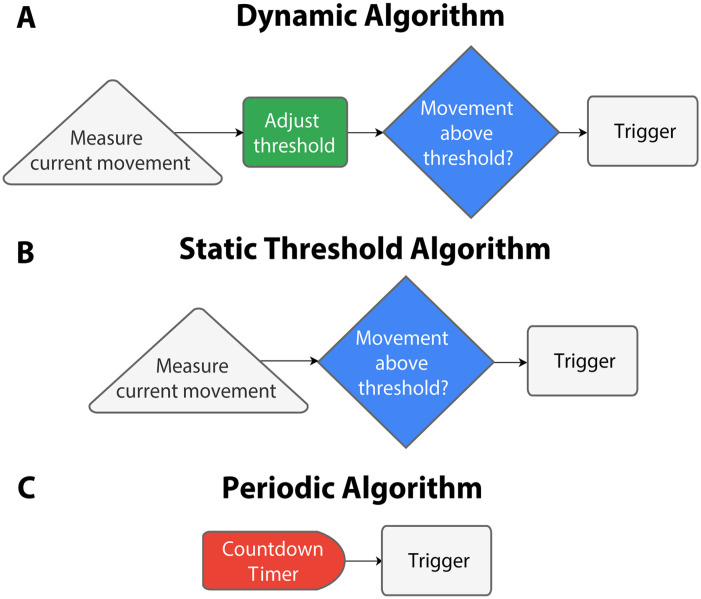
Graphical description of each algorithm. (A) The dynamic algorithm measures the movement in progress and adjusts a minimum activity level and triggers stimulation if that movement exceeds the threshold. (B) The threshold algorithm measures the movement in progress and triggers stimulation if that movement is larger than a preset minimum activity level. (C) The periodic algorithm employs a countdown timer that triggers stimulation upon expiration.

**Figure 3.**
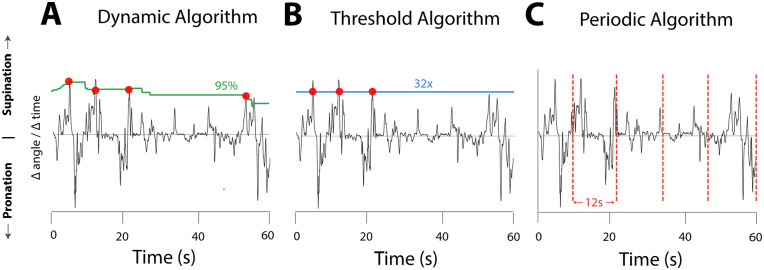
Examples of triggering during rehabilitative exercises with each algorithmic method. A rotation exercise produced the representative signal in each plot. (A) The dynamic algorithm triggers stimulation when movement crosses a variable threshold based on percentile of recent movement. The green curve represents a threshold that mark the 95th percentile of recent movement in the direction of supination. (B) The threshold algorithm triggers stimulation when movement crosses beyond preset movement levels. The blue horizontal line represents the preset movement level, which is set to 32× the movement minimum in this example. (C) The periodic algorithm triggers stimulations every 12 seconds, regardless of movement. Vertical dashed lines represent VNS stimulations. Red dots represent the movement level that coincided with the VNS trigger.

We developed 3 algorithms premised on differing selection criteria and applied them to the previously collected data. The first algorithm delivered stimulation triggers when the movement signal exceeded a dynamically-adjusted minimum activity threshold, which varied during the exercise session based on recent movement (Figures 2A and 3A). The second algorithm delivered stimulation triggers when the movement signal exceeded a fixed minimum activity threshold (Figures 2B and 3B). The third algorithm delivered stimulation triggers at a regular interval, irrespective of the movement signal (Figures 2C and 3C). To evaluate the performance of these algorithms, we applied each algorithm to the previously collected data sets and examined movement magnitude and interval between triggering instances. Additionally, we compared the performance of the dynamic algorithm to previously recorded triggering selections made by a therapist during rehabilitation exercises.

### A Dynamic Algorithm Pairs VNS With the Best Movements and Produces Minimum Triggering Rate Variability

**Figure 4.**
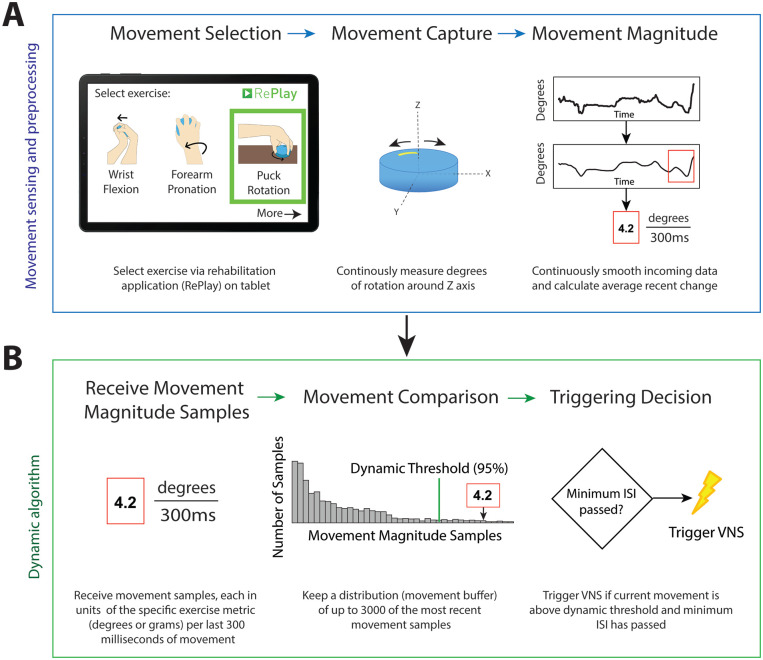
Illustrative example of movement sampling and the processes employed by the dynamic algorithm. (A) Prior to analysis by the algorithm, the movement signal is collected by the sensor and preprocessed. Step 1: Users select specific exercises from the RePlay application to guide the rehabilitation and select the relevant sensor dimension. Step 2: Sensors capture movement in a single relevant dimension while the rehabilitation exercise is performed. Step 3: The movement data is continuously smoothed with a convolution filter to remove the noise. The gradient of the samples in the last 300 ms of the smoothed signal produces the rate of change. The preprocessing ends by calculating the average rate of change within the 300 ms window. (B) Preprocessed movement signal samples are continuously delivered to the algorithm for triggering decisions. Step 1: The algorithm receives single values previously calculated from the average rate of change in the movement signal. Step 2: The dynamic algorithm identifies the size of the current movement sample by its location in the distribution of up to 3000 recent samples. Step 3: If the movement magnitude is in the top 5% of the 3000 samples, the algorithm triggers VNS if the 5 s minimum inter-stimulus interval has passed.

**Figure 5.**
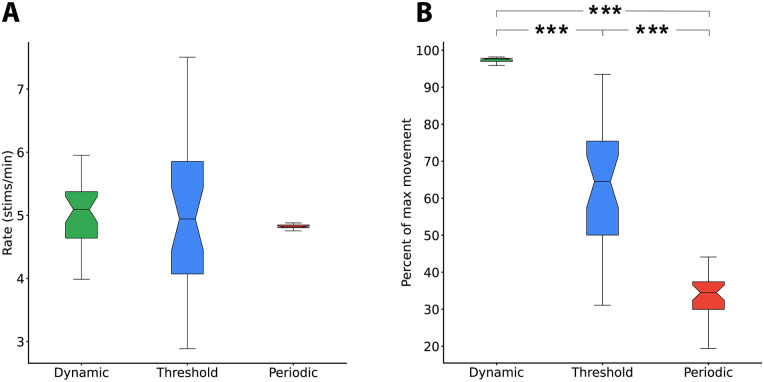
The dynamic algorithm yields the most robust selection of large movements and a reliable triggering interval. (A) The periodic algorithm produced consistent triggers every 12 seconds for a rate of 5 stims/minute. Variance of the periodic triggering rate seen here is from gameplay times not divisible by 12. The static threshold algorithm produced a median triggering rate of 4.94 (IQR = 1.78) stimulations per minute with considerable variance. The dynamic algorithm produced a median triggering rate of 5.09 (IQR = 0.74) stimulations per minute with moderate variance. No significant differences between median rates were observed. The notched boxes in each plot represent 1160 exercise sessions and include all controllers, games, and participants. Notches represent a 90% CI of the median. (B) The quality of the movements selected by the algorithms are represented as the percent of maximum movements during each exercise. The periodic algorithm triggered VNS on 34.50% (IQR = 7.47) of maximum movement. The threshold algorithm triggered VNS on 64.50% (IQR = 25.38) of maximum movement. The selective algorithm triggered VNS on 97.61% (IQR = 0.88) of maximum movement.

First, we explored the performance of a dynamic algorithm. Dynamic algorithms that select the best trials per subject during rehabilitation have consistently been employed during preclinical studies involving rehabilitation of force and range of motion.^9,20-23^ Since variability of impairment levels between stroke or SCI patients is a consideration, the dynamic algorithm was designed to actively adjust the stimulation threshold to account for differences in performance across subjects and exercises (Figure 4). We examined the algorithm at multiple minimum activity thresholds created from distributions of recent movement (Percentiles: 45%, 55%, 65%, 75%, 85%, and 95%). Analysis of the algorithm showed VNS was routinely paired with movements above the set minimum percentile of recent movement. When the algorithm was set to pair VNS with movements above the 95th percentile of recent movements, the algorithm triggered at 5.09 (IQR = 0.74) stimulations per minute and selected movements at a percentile of 97.61% (IQR = 0.88) during VNS (Figure 5A and 5B).

All triggers were paired with movements above the 95th percentile of recent movements. Moreover, the dynamic algorithm produced triggers in all exercise samples, indicating that this approach is able to adjust to various types of signals and levels of participant performance.

### A Static Threshold Algorithm Pairs VNS With Movements But Produces Substantial Triggering Rate Variability

Second, we evaluated the performance of a static threshold algorithm. The static threshold reduced the complexity of the signal processing, but as a tradeoff, it could reduce the flexibility to accommodate differences in performance across different patients and exercises. We explored the performance of this algorithm at multiple fold increases over the noise floor (Movement minimum multipliers = 1, 2, 4, 8, 16, and 32). For comparison, we set the level that produced a median triggering rate similar to the dynamic algorithm. Figure 5A and 5B shows a movement minimum multiplier of 32× produces a triggering rate of 4.94 (IQR = 1.78) stimulations per minute and selectivity of 64.49% (IQR = 25.38).

29.03% of sessions resulted in no stimulation triggers during the entirety of the session. Individual analyses of these sessions revealed that participants were moving during the session, but the movements were not large enough to surpass the static threshold. About 43.86% of sessions resulted in total movement-VNS pairings below 95% selectivity. Individual analyses of these sessions revealed an abundance of activity above the minimum activity threshold that was set. As expected, when the triggering threshold was increased, the selectivity of the algorithm increased, and the triggering rate decreased (Supplemental Figure 1). At high thresholds, selection emphasized performance-based triggering, so stimulations only occurred on the largest movements. However, this consequently produced less frequent triggers. Similarly, when the triggering threshold was decreased, the selectivity of the algorithm decreased and the triggering rate increased.

### A Periodic Algorithm Provides Consistent Inter-Stimulation Intervals But Poor Selectivity

Finally, we investigated the performance of a periodic algorithm. This is advantageous in that it represents the simplest implementation of signal processing, but because it does not expressly account for performance, it may fail to effectively trigger stimulation concurrent with the best movements. We evaluated performance at multiple inter-stimulus intervals (6, 6.67, 7.5, 10, 12, and 15 seconds). By design of the algorithm, each timing parameter resulted in a consistent stimulation interval at the set value (Figure 5A). Because this approach does not consider movement when determining triggering, the algorithm often produced trigger events when no movement was occurring and only rarely produced trigger events during large movements. As a result, the algorithm consistently triggered during movements that were below the 50th percentile of recent movements. The median movement selectivity was 34.05% (IQR = 7.47) when the inter-stimulus interval was set to 12 seconds (Figure 5A and 5B). Because the algorithm does not account for movement, it frequently triggered during periods of rest, which decreased the percentile of the selected movements. When the inter-stimulus interval was shortened or lengthened, the selection characteristics of the algorithm do not improve (Supplemental Figure 1). Thus, the periodic algorithm produced reliable trigger intervals, but demonstrated poor selectivity for the best movements.

### Not All Algorithms Create Appropriate Triggering Intervals

The periodic and dynamic algorithms can maintain a desired triggering interval while providing 5 triggers per minute (12 seconds between triggers), which matches the rate used in previous studies that demonstrate VNS-dependent benefits.^4-6^ In the periodic algorithm, the inter-stimulus interval is the only input parameter and is constant across exercise types and patients (Figure 5B). The threshold and dynamic algorithms employ a minimum inter-stimulus interval to ensure stimulations are separated by at least 5 seconds. When the minimum activity threshold in the static algorithm is set to 32 times the movement minimum, the mean inter-stimulus interval is 27.16 ± 2.01 seconds for sessions where triggering occurred. When the minimum percentile of recent movement is set to 95% in the dynamic algorithm, the average inter-stimulus interval is 13.87 ± 0.22 seconds. Thus, the periodic and dynamic algorithms can produce triggering near the desired 12-second interval but the threshold algorithm does not consistently produce enough triggers.

### The Dynamic Algorithm Maximizes Movement Magnitude Across All Exercises and Capabilities

**Figure 6.**
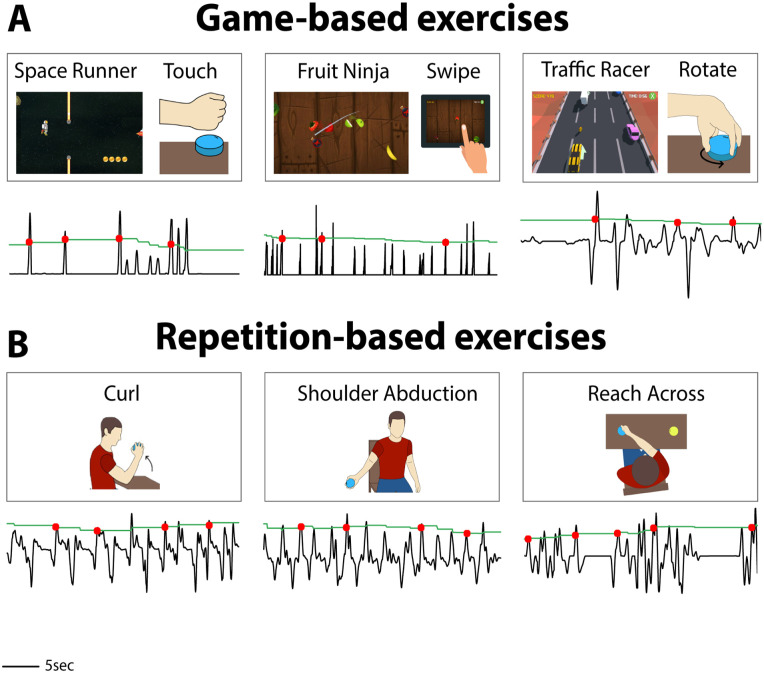
Illustrative examples of exercise signals after processing by the dynamic algorithm. Each processed signal is labeled with its game title and exercise type. Puck movements were translated to rate of change signals. Swiping speed was extracted during gameplay and used as the main signal for Fruit Ninja. Red dots indicate movement instances that triggered VNS. The dynamic algorithm triggers a stimulation when movement crosses an active threshold, represented as a percentile of recent movement. The green curve represents a threshold that marks the 95th percentile of recent movement (last 3000 samples).

Triggering stimulation to coincide with the best movements during rehabilitation is necessary for VNS-dependent benefits.^24^ We compared the selectivity of the algorithms to determine which algorithm balanced consistent timing with triggering on the best movements. The quality of the movements selected by the algorithms are represented as the percent of maximum movements during each exercise. Overall, the dynamic algorithm resulted in the greatest percent of maximum movement compared to periodic and static algorithms (Figure 5B, periodic: 33.86 ± 1.01%, paired t-test, *P* = 1.13 × 10^−34^; static: 64.30 ± 3.04%, paired *t*-test, *P* = 2.77 × 10^−12^). This indicates that the dynamic algorithm provides the most reliable selection of the best movements across exercises and participants (Figure 6, Supplemental Table 1).

### The Dynamic Algorithm Selects Larger Movements Than Supervised Manual Triggering

**Figure 7.**
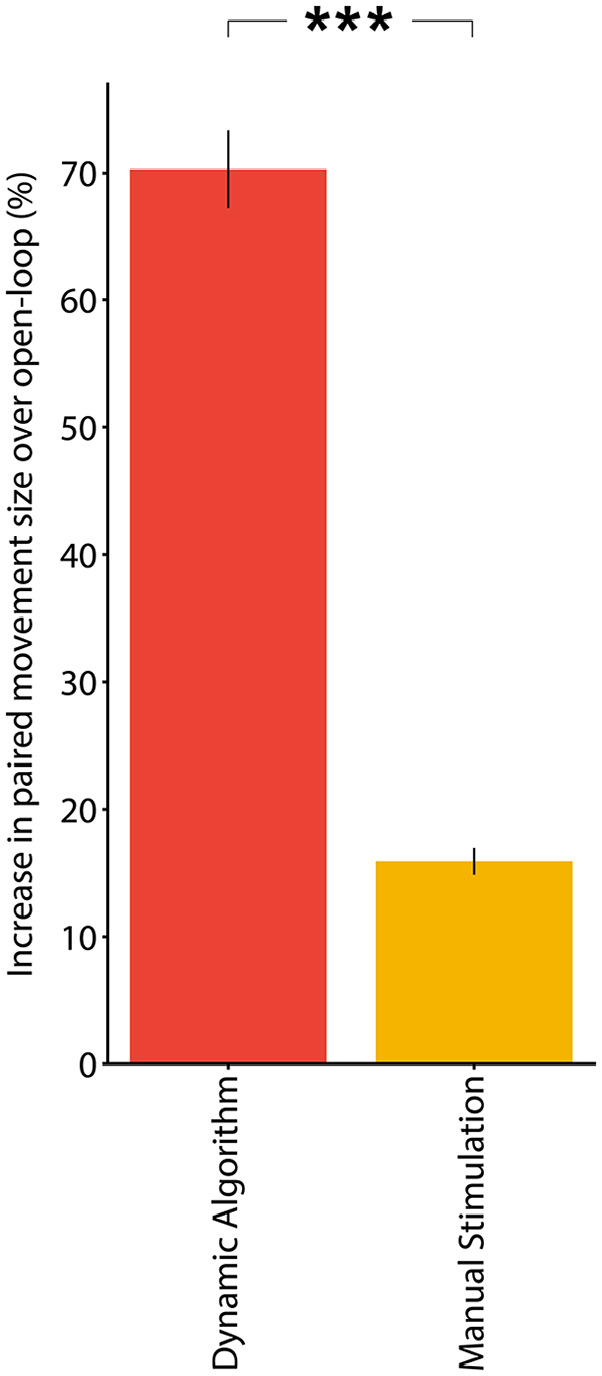
The dynamic algorithm selects larger movements than a trained observer. During upper limb physical therapy with RePlay and ReCheck, the dynamic algorithm triggered stimulation on movements that were 54.38 ± 2.97% larger than movements selected by a trained physical therapist (unpaired 2-tailed *t*-test, *P* = 1.77 × 10^−74^). We individually normalized the movement data by calculating the average paired peak size within ± 1 second of periodic stimulations at 12 second intervals throughout the therapy session. The dynamic algorithm and the periodic algorithm were applied in post-hoc analysis and the manual stimulations were conducted in real time.

Based on the ability to trigger at the desired interval and selection of the largest movements, the dynamic algorithm represented the optimal triggering paradigm of those tested. Because the algorithm is ultimately intended to facilitate the delivery of VNS therapy by reducing the burden on a therapist to trigger stimulation, we sought to directly validate performance by comparing to dynamic algorithmic stimulation selection to that delivered by a trained therapist. To do so, we reanalyzed a large set of rehabilitative data in which a therapist triggered stimulation and compared the paired movement magnitude and stimulation timing to the dynamic algorithm’s selections. We individually normalized the movement data by calculating the average paired peak size within ±1 second of periodic stimulations at 12 seconds intervals throughout the therapy session, for all possible 2-second samples. Normalization was performed per participant, per exercise, per game, for each therapy date. The selection quality of the manual and dynamic algorithm triggers is represented by the percent improvement of the paired movement peaks over the periodic algorithm. Both manual triggers and the dynamic algorithm pair VNS with larger movement peaks than the periodic algorithm. Peak-pairing performance of the dynamic algorithm indicates that the algorithm selects large movements at least as well as a trained human observer (Figure 7, *P* = 1.77 × 10^−74^, dynamic algorithm stimulations: 25 203, manual stimulations: 31 079). On average, the dynamic algorithm selects movements that are 70.28 ± 3.03% bigger than the periodic algorithm and 54.38 ± 2.97% larger than therapist-selected movements (Figure 7).

## Discussion

Here, we report the design of an algorithm capable of pairing VNS with the best movements during upper limb rehabilitation following neurological injury. We recorded movement data from 14 stroke and 18 SCI patients during a variety of different rehabilitative exercises. We used this data to develop a dynamic algorithm and compare it to alternative and clinically-employed algorithms to compare which strategy exhibits the best triggering criteria. After testing a range of parameters within each of the algorithms, we identified a set of parameters within the dynamic algorithm that can select the best movements while maintaining a consistent median triggering rate. Additionally, we validated that the dynamic algorithm performs at least as well as a trained human observer, indicating that this approach represents a means to provide unsupervised, closed-loop VNS during rehabilitation.

The motivation for this study was to develop an algorithm to facilitate feedback-controlled neurostimulation during rehabilitation, aimed at increasing the dose and quality of VNS pairings. Previous studies show stimulation timing and trial selection affect the magnitude of VNS-dependent enhancement of post-stroke and post-SCI recovery.^8,9,20,24,25^ A recent preclinical study clearly illustrates the reliance of VNS effects on trial selection. Pairing VNS with the strongest forelimb movements during rehabilitative training significantly enhanced recovery of forelimb strength, whereas pairing the weakest movements failed to promote recovery.^9^ Pairing VNS with an adaptive threshold algorithm in models of stroke, SCI, and peripheral nerve injury resulted in significantly enhanced forelimb recovery.^9,20-23^ Moreover, several studies confirm that a matched amount of stimulation that is not paired with movement fails to enhance recovery.^6,8,25^ These provide the rationale for developing an algorithm that can trigger stimulation concurrent with the best movements. Several additional lines of evidence demonstrate the importance of stimulation timing on VNS-dependent effects.^26^ Faster rates of stimulation (ie, shorter inter-stimulation intervals) are associated with smaller VNS-dependent effects in preclinical models.^27,28^ Moreover, clinical evidence shows large amounts of periodic VNS during rehabilitation provides only modest benefits compared to stimulation delivered explicitly concurrent with exercises.^5,10^ Together, these findings reinforce the importance of incorporating inter-stimulation timing into an algorithm for unsupervised stimulation. Overall extraction of these findings indicates that VNS is likely most effective when therapy sessions include an effective number of stimulations that incorporate the best movements with the longer intervals between stimulations.

Given the reliance on movement selection and inter-stimulation timing, we developed algorithms with different characteristics to achieve VNS triggering based on these factors, including replicating the methods that were effective in preclinical studies. Studies indicate that timing VNS delivery with a dynamic threshold algorithm that adaptively scaled on the median peak force of the 10 antecedent trials resulted in significantly more recovery than unpaired VNS or sham stimulation.^9,20-23^ Successful translation of this approach from bench to clinic requires task consideration. Patients perform continuous tasks while playing games for several minutes at a time to maximize repetitions within the allotted rehabilitation session time. Thus, the dynamic algorithm employed here was augmented to fit a continuous signal while still adjusting the VNS threshold based on recent movement.

Each of the algorithms explored here has unique benefits. The periodic algorithm employs simple timing for the sake of prioritizing the rate of VNS over the quality of its pairings and matches the conventional method for unsupervised VNS delivery. This algorithm is beneficial for ease of implementation and simplifies validation testing. However, since the periodic algorithm is operated by a countdown timer, it does not account for movement quality and thus does not provide selection of the best movements. Alternatively, the static algorithm can select movements while maintaining an optimum stimulation rate. The capability to select movements makes a static threshold appealing, but the realistic application of such an algorithm is hindered by the large variability of impairment levels observed in patients with neurological injuries. Additionally, motor performance can fluctuate day-to-day or with improvement over the course of rehabilitation, which complicates selection of an appropriate static threshold. If the threshold is set too high, a patient may not perform any movements of a magnitude great enough to trigger VNS. If the threshold is set too low, movement of virtually any magnitude will trigger stimulation, which limits selection of the best movements. The dynamic algorithm compensates for this issue by automatically individualizing the triggering criteria in real-time without the need to fine tune parameters for each person or session. An adaptive minimum activity threshold fluctuates according to recent movement to achieve selectivity on the best movements while maintaining the optimum rate of stimulation. The dynamic threshold ensures the algorithm can be applied to various exercises across a range of impairment levels without losing its main advantages. These characteristics increase the number of ideal VNS pairings during therapy.

VNS has emerged as an FDA-approved strategy to enhance rehabilitation following neurological injury. Previous studies show that manually triggered VNS paired with upper limb rehabilitation can reduce long-term deficits following stroke.^4-6^ In its conventional implementation, VNS is delivered by a therapist who pushes a button during movements they want to reinforce. This method enhances motor function and improvements persist over time, demonstrating that supervised rehabilitation with VNS can generate long-lasting, clinically significant improvements in stroke patients. However, patients that receive VNS with rehabilitation still exhibit residual deficits.^4-6^ Maximizing the clinical impact of VNS for stroke recovery may depend on the selection of an algorithm that can properly address many unique movements that take place during rehab. Some clinical observations support this notion. Whereas patients demonstrated significant changes in function when VNS was triggered by a therapist observing movement, patients from a pilot study demonstrate comparatively modest gains when receiving unsupervised VNS that was not explicitly paired with movements, even when stimulation was delivered for years.^5,10^ This approach uses a stimulation paradigm congruent to the periodic algorithm described in this study. The absence of continued improvements in function may reflect the lack of consistent stimulation during the best movements. Given the advantage in selectivity with the dynamic algorithm, it is reasonable that using this approach to deliver unsupervised closed-loop stimulation during rehabilitation over a long time-course may represent a means to drive greater recovery.^16,28^

Since the conventional approach involves a therapist pairing VNS manually with rehabilitative movements, we sought to determine if the dynamic algorithm could select movements at least as well as a trained observer. An algorithm capable of matching the selection characteristics of a human observer would allow for paired VNS during unsupervised rehabilitation and also let therapists focus on rehabilitative exercises rather than stimulation timing. In the current study, therapists observed patients during gaming and pressed a button to deliver stimulation when large movements were observed, with a limit of at least 5 seconds between consecutive stimulation. We used this manual stimulation timing data to conduct post-hoc timing analysis of the dynamic algorithm and the human observer on the same movements. The result of this comparison indicates that the algorithm is able to match and exceed the selection of large movements during therapy. The automation of this effective conventional approach indicates the algorithm could be used for unsupervised at-home VNS.

Full automation of movement selection must consider unbalanced deficits that could be present during bidirectional exercises. Motor impairments after neurological injury commonly present as deficits that exist to a greater degree in 1 direction over another, such as a moderate impairment of forearm supination and severe impairment of forearm pronation in the same arm. These deficits appear as low levels of activity in 1 direction of the respective movement signal. We considered this scenario and designed the dynamic algorithm to handle bidirectional movements by optionally maintaining 2 separate movement distributions, which provides automatic adjustment of 2 individual thresholds during bidirectional movements (Supplemental Figure 2). The static algorithm can compensate when different minimum activity thresholds are set for each direction, but extensive manual tuning would be needed. The periodic algorithm is unable to compensate for a deficit imbalance, indicating it is least suitable for handling bidirectional movement training.

This algorithm is designed to identify instances where a single-dimension signal outperforms its own previous activity; however, greater complexity of movement analysis that require multidimensional examination may be valuable, such as during bimanual exercises. In the future, signal preprocessing could combine multiple dimensions by averaging multiple channels together, taking the largest channel, or many other methods that could be tested and employed. Future implementations could utilize multiple signals competitively to prevent stimulation during movement with characteristics that would suggest unwanted compensation.

In addition to developing an algorithm that can select optimal movements while maintaining an effective triggering rate, the simplicity of the algorithm is a consideration. It is likely that more complex methods, such as approaches premised on machine learning, could be comparable or superior at movement selection. However, we sought to develop an algorithm that yielded the appropriate behavior with minimal complexity based on 2 overarching considerations. First, machine learning algorithms can be computationally intensive, and we sought to avoid a scenario that required combining a high bandwidth computation algorithm and the software that governs control of the VNS system on the same smart device. Second, the black-box nature of machine learning complicates verification and validation testing for regulatory approval, a crucial consideration in eventual deployment of this approach. We expect this algorithm to be useful in future applications of medical devices with physiologic closed-loop control technology or for integration into Software as a Medical Device; subsequently, we have followed all relevant and emerging guidance, such as simplicity of system integration and operational transparency for clinicians, while meeting our requirements for rate and signal peak selection.

Here, we describe an algorithm that can be used to pair VNS with the best movements at a reliable interval to replicate and build on the manually paired VNS delivery paradigm that produced clinical benefits. Automatic closed-loop VNS can be achieved with an algorithm that dynamically modulates a minimum activity threshold based on previous movements. This approach performs at least as well as a trained human observer, providing initial evidence of validity. If effective, this strategy could improve the timing of VNS delivery during rehabilitation, reduce on therapists of simultaneously overseeing rehabilitative exercises and stimulation delivery, and allow for closed-loop unsupervised stimulation at home to extend the duration of therapy. Future studies should implement this algorithm to control VNS delivery and determine whether this approach can complement conventional VNS therapy to generate greater recovery in individuals with neurological injury. The algorithm may also be effective in providing closed-loop neuromodulation via transcranial magnetic stimulation, spinal cord stimulation, cortical stimulation, deep brain stimulation, or peripheral nerve stimulation.

## Supplemental Material

sj-docx-1-nnr-10.1177_15459683241252599 – Supplemental material for Characterization of an Algorithm for Autonomous, Closed-Loop Neuromodulation During Motor Rehabilitation Supplemental material, sj-docx-1-nnr-10.1177_15459683241252599 for Characterization of an Algorithm for Autonomous, Closed-Loop Neuromodulation During Motor Rehabilitation by Joseph D. Epperson, Eric C. Meyers, David T. Pruitt, Joel M. Wright, Rachael A. Hudson, Emmanuel A. Adehunoluwa, Y-Nhy Nguyen-Duong, Robert L. Rennaker, Seth A. Hays and Michael P. Kilgard in Neurorehabilitation and Neural Repair
