# Supplementary material for: Characterization of an Algorithm for Autonomous, Closed-Loop Neuromodulation During Motor Rehabilitation
Source: Neurorehabil Neural Repair. 2024 May 7;38(7):493–505. doi: 10.1177/15459683241252599 (PMC11179975; doi:10.1177/15459683241252599)
Supplement: sj-docx-1-nnr-10.1177_15459683241252599 – Supplemental material for Characterization of an Algorithm for Autonomous, Closed-Loop Neuromodulation During Motor Rehabilitation [file sj-docx-1-nnr-10.1177_15459683241252599.docx]

Supplemental Materials

*Supplementary Table 1: Characteristics and results of tested algorithms.*

| **Algorithm** | **Automatically pairs triggers with movement?** | **Automatically handles variability?** | **Stimulation Rate (Median stim/min)** | **Triggering Selection (% of max movement)** |
| --- | --- | --- | --- | --- |
| Dynamic threshold | Yes | Yes | 5.09 (IQR: 0.74) | 97.61 (IQR 0.88) |
| Static threshold | Yes | No | 4.94 (IQR: 1.78) | 64.49 (IQR 25.38) |
| Periodic triggering | No | No | 5* | 34.05 (IQR 7.47) |

*Periodic algorithm was set to trigger every 12 seconds

**
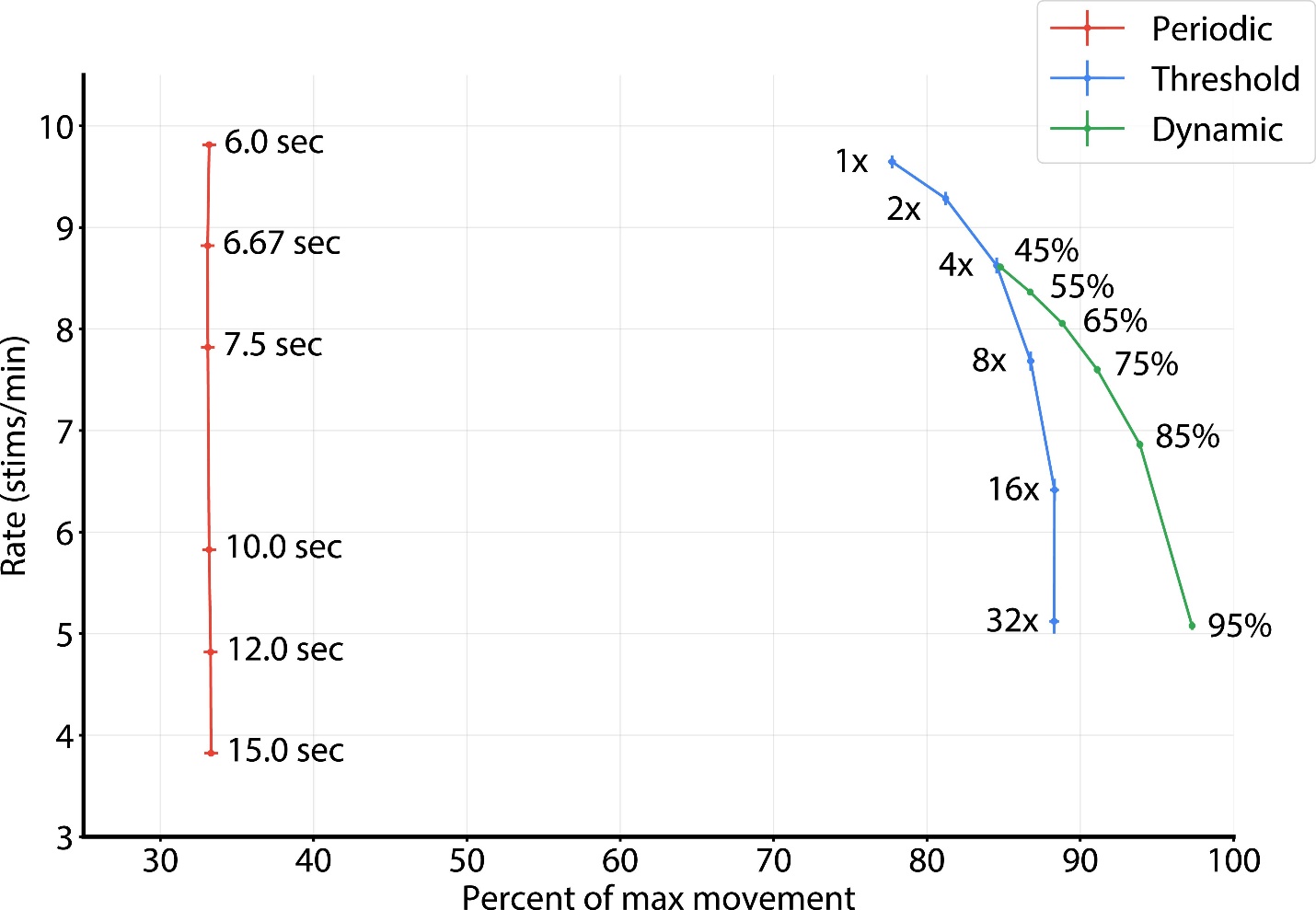
**

**Supplementary Figure 1: The dynamic algorithm yields superior triggering characteristics following parameter sweeping analysis.** Input parameters for each algorithm were varied to observe their effect on triggering rate and movement selection quality. All parameters were held constant except for those notated in the figure. The periodic algorithm yielded triggering rates that closely matched its chosen interstimulus intervals and did not select trials over 35% of max movement. Triggering rate and trial selection varied as a function of the movement minimum multiplier of the static threshold algorithm. Small movement minimum multipliers caused the threshold algorithm to select small movements and high triggering rates, while large movement minimum multipliers trended toward selecting large movements and low triggering rates. Varying the selected percentile of recent movement allows the dynamic algorithm to vary its minimum activity threshold. Triggering rate and trial selection quality varied while sweeping the minimum activity threshold of the dynamic algorithm. Low minimum activity thresholds caused the dynamic algorithm to select small movements and high triggering rates, while high minimum activity thresholds yielded the selection of large movements and low triggering rates. The dynamic algorithm selected larger movements than the periodic and static threshold algorithm at the same triggering rates.

**
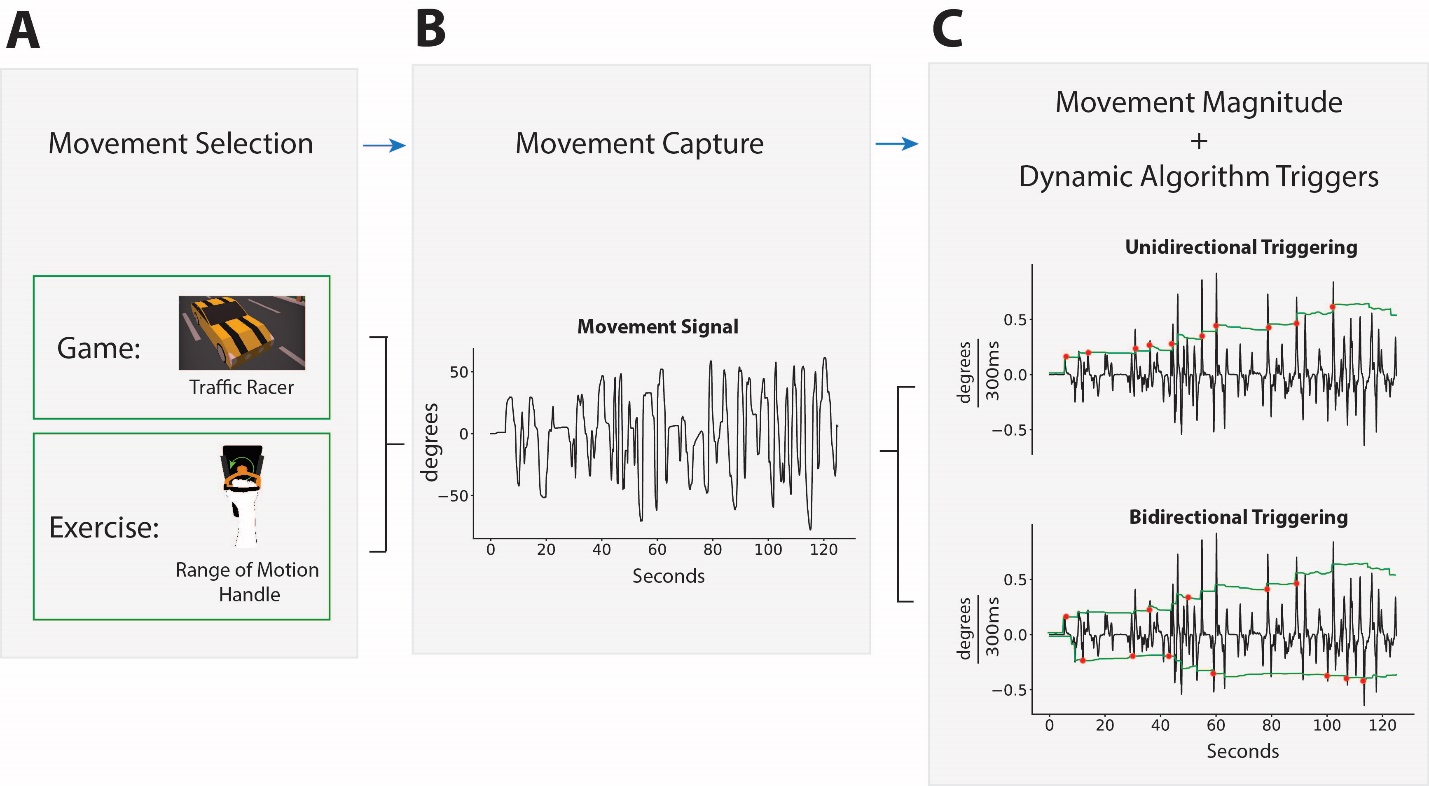
**

**Supplementary Figure 2: The dynamic algorithm can support both unidirectional and bidirectional triggering.** Users can set the dynamic algorithm to trigger VNS based on a single movement distribution (unidirectional) or two separate movement distributions (bidirectional). Unidirectional triggering may be preferred when rehabilitation is focused on recovering range of motion or strength in a single dimension, while bidirectional triggering may be preferred when targeting general increases in range of motion or strength. This option provides flexibility to handle VNS timing in patients with unbalanced deficits. (A) Users select the game and exercise in the rehabilitation application (RePlay) to isolate the signal in the dimension(s) of interest, for example, Traffic Racer controlled with the Range of Motion Handle exercise. (B) The hardware and software capture the pronation and supination movements in degrees during the rehabilitative exercise. The application continuously preprocesses the signal to extract the rate of change of the movements for the algorithm process. (C, top) The unidirectional triggering setting for the dynamic algorithm can be set to Positive Only or Negative Only. Here, the Positive Only setting shows the green dynamic threshold produces triggers only when movements surpass the 95^th^ percentile of supination movements. Red dots indicate movements that produced triggers (C, bottom) The bidirectional triggering setting for the dynamic algorithm produces triggers when the movement surpasses either the 95^th^ percentile of either the independent supination or pronation thresholds, shown in green. Red dots indicate movements that produced triggers. Minimum interstimulus interval is set to 5 seconds for both examples.
